# Cyanobacterial harmful bloom lipopolysaccharides: pro-inflammatory effects on epithelial and immune cells in vitro

**DOI:** 10.1007/s00204-023-03644-8

**Published:** 2023-12-08

**Authors:** V. Skočková, P. Raptová, K. Pospíchalová, I. Sovadinová, E. Sychrová, M. Smutná, K. Hilscherová, P. Babica, L. Šindlerová

**Affiliations:** 1 Department of Biophysics of Immune System, Institute of Biophysics of the Czech Academy of Sciences, Brno, 61200 Czech Republic; 2 Department of Experimental Biology, Faculty of Science, Masaryk University, Brno, 62500 Czech Republic; 3 RECETOX, Faculty of Science, Masaryk University, Brno, 62500 Czech Republic; 4 Department of Experimental Phycology and Ecotoxicology, Institute of Botany of the Czech Academy of Sciences, Brno, 60200 Czech Republic

**Keywords:** Lipopolysaccharide, Cyanobacterial harmful blooms, Inflammation, Keratinocytes, Enterocytes, Immune cells

## Abstract

Cyanobacterial harmful blooms (CyanoHABs) pose a global ecological problem, and their lipopolysaccharides (LPS) are among the bioactive compounds they release. Previous studies on CyanoHAB-LPS from single cyanobacterial species have shown varying bioactivities in different in vitro cell models. In this study, we isolated LPS from 19 CyanoHAB samples collected at 18 water bodies in the Czech Republic over two consecutive seasons. The proportions of cyanobacteria, Gram-negative bacteria (G-), and other bacteria in the biomass were determined by qPCR, while the cyanobacterial genera were identified using light microscopy. In vitro models of keratinocytes (HaCaT), the intestinal epithelium (co-culture of differentiated Caco-2 cells and peripheral blood mononuclear cells — PBMC), and PBMC alone were treated with isolated LPS at concentrations of 50, 100, and 1 µg/ml, respectively. The endotoxin activities of these concentrations were within the range measured in the aquatic environment. Approximately 85–90% of the samples displayed biological activity. However, the potency of individual LPS effects and response patterns varied across the different in vitro models. Furthermore, the observed activities did not exhibit a clear correlation with the taxonomic composition of the phytoplankton community, the relative share of microbial groups in the biomass, endotoxin activity of the LPS, or LPS migration and staining pattern in SDS-PAGE. These findings suggest that the effects of CyanoHAB-LPS depend on the specific composition and abundance of various LPS structures within the complex environmental sample and their interactions with cellular receptors.

**Supplementary Information:**

The online version contains supplementary material available at 10.1007/s00204-023-03644-8.

## Introduction

The occurrence of cyanobacterial harmful blooms (CyanoHABs) in freshwater reservoirs is a global environmental issue (Huisman et al. 2018). Cyanobacteria are capable of producing a wide range of hazardous toxins, mostly secondary metabolites, some of which, such as microcystin or cylindrospermopsin, are well-studied and characterized. Their production can lead to adverse health effects, including fatalities (Buratti et al. 2017). However, there are also bioactive and potentially toxic cyanobacterial secondary metabolites, like puwainaphycins and minutissamides, for which limited information exists regarding their environmental occurrence, fate, effects, and associated risks (Huisman et al. 2018; Kubickova et al. 2019; Vašíček et al. 2020). CyanoHABs also represent a source of endotoxins/lipopolysaccharides (LPS). LPS form an integral part of the outer membrane of the cell wall in cyanobacteria and heterotrophic Gram-negative (G-) bacteria which associate with CyanoHABs (Caroff and Novikov 2020; Gemma et al. 2016). Consequently, LPS is inherently present in any location with CyanoHAB occurrence, where it may be released into the water during cell division and lysis (Durai et al. 2015; Sulc et al. 2017).

Some cases of recreational exposure to CyanoHABs leading to skin irritation, gastroenteritis, and other adverse inflammation-based effects were previously published (Kubickova et al. 2019), and also our previous results indicate the pro-inflammatory potency of several CyanoHAB-derived LPS and ability to potentiate the effect of cylindrospermopsin. However, these studies were primarily focused on *Microcystis aeruginosa* (Bláhová et al. 2013) or on a limited number of CyanoHAB biomasses heavily dominated by a selected cyanobacterial species of interest (Moosova et al. 2019; Moosová et al. 2019; Raptová et al. 2023; Skočková et al. 2023). Consequently, these studies may not sufficiently represent the variability of potencies and activities of CyanoHAB-derived LPS across a broader spectrum of naturally occurring water blooms with different dominants and species composition. Therefore, in the present study, we collected 19 CyanoHAB biomasses from 18 recreational water bodies in the Czech Republic during two consecutive seasons to more comprehensively characterize the diversity of CyanoHAB-LPS, their endotoxin activities, and biological effects on cellular in vitro models of keratinocytes (HaCaT cells), enterocytes (differentiated Caco-2 cells) co-cultured with human peripheral blood mononuclear cells (PBMC), and PBMC themselves.

## Methods

Detailed specifications of used buffers, media, antibodies, and kits please find in the Supplementary Material.

### CyanoHAB samples

The preparation of the CyanoHAB biomasses collected from water reservoirs in the Czech Republic is described in Skočková et al. (2023). The phytoplankton cell counts and species composition were determined using microscopic evaluation of sample aliquots fixed with 2% formaldehyde. The cell counts were converted to biovolumes (Hillebrand et al. 1999; Skácelová and Lepš 2014) and expressed as a percentage share of biovolume in a particular sample. The sample codes, dominant groups and species, biovolume percentages for each sample, and identification of sampled localities are summarized in Supplementary Table S1.

### LPS preparation and electrophoretic separation

The extraction of CyanoHAB-LPS was performed using a hot phenol−water extraction method, as previously described (Skočková et al. 2023). Isolated and purified LPS were separated by SDS-PAGE followed by silver or Pro-Q^™^ Emerald 300 staining. Protein contamination was evaluated using SDS-PAGE and Sypro Ruby staining, nucleic acid contamination by agarose gel electrophoresis and ethidium bromide staining. For procedure details see the Supplementary Materials.

### DNA extraction

The extraction of DNA from CyanoHAB biomasses was conducted based on previous studies (Sehnal et al. 2022; Skočková et al. 2023). The details of the procedure are described in Supplementary Materials.

### qPCR detection of cyanobacteria, heterotrophic bacteria, and Gram-negative heterotrophic bacteria

DNA quantification was performed using qPCR and specific FAM-BHQ1-labeled Taqman probes and primers that targeted 16S rRNA genes as reported previously (Skočková et al. 2023). The primers and probes (Supplementary Table S2) were designed to detect the gene sequences specific for three groups of microorganisms: cyanobacteria, total heterotrophic bacteria, and a subset of G- heterotrophic bacteria. The qPCR was conducted on a LightCycler^®^ 96 Real-time PCR System under the same conditions as reported in a previous study (Lang-Yona et al. 2014). qPCR values (copies of respective 16S rRNA genes) were converted to cell equivalents using a calibration curve constructed using a cultured *E. coli* CCM 3954 (for total and G- bacteria) or *Microcystis aeruginosa* PCC7806 (for cyanobacteria). The obtained cell count equivalents were normalized per total isolated DNA, and expressed as a percentage share of each group, with the sum of cyanobacteria and total heterotrophic bacteria considered as 100%. The share of heterotrophic bacteria other than G- bacteria was determined by subtracting G- bacteria from the total heterotrophic bacteria.

### Endotoxin activity by PyroGene^™^ rFC assay

The endotoxin activity of isolated LPS was determined using the PyroGene^™^ Recombinant Factor C Endpoint Fluorescent Assay (Lonza, Switzerland) following the manufacturer’s instructions, as described previously (Skočková et al. 2023). Fluorescence was measured using a microplate reader (Infinite M200, Tecan, Switzerland; excitation 380 nm, emission 440 nm), and endotoxin activity was calculated according to the standard curve. Data were expressed as endotoxin activity in endotoxin units (EU) per g of biomass dry weight (d. w.) and per mg of the respective LPS, endotoxin activities of CyanoHAB-LPS concentrations used in in vitro experiments were calculated (Table S3).

### Cell lines

Caco-2 cells (human colorectal adenocarcinoma cell line; CLS, Germany) were maintained in the Caco-2 medium. The cells were seeded at a density of 2 × 10^4^ cells/cm^2^ on 24-well cell culture inserts (Corning, USA) and maintained for 21 days to reach a differentiated monolayer. The apical (300 μl) and basolateral (800 μl) culture media were changed three times per week.

HaCaT cells (human keratinocyte cell line; CLS, Germany) were maintained in HaCaT medium. The cells were seeded at a density of 4 × 10^4^ cells/cm^2^ on a 24-well or 96-well plate (TPP, Switzerland) and maintained for 3 days to reach a confluence.

### PBMC isolation

Peripheral blood from healthy donors was obtained based on the collection protocol approved by The Ethics Committee for Research at Masaryk University, Brno, Czech Republic (number EKV-2018-083). The PBMC isolation procedure was based on the work of Meital et al. (2019). The detailed procedure is provided in the Supplementary Materials.

### Treatment

HaCaT cells were treated with 50 μg/ml of CyanoHAB-LPS, Caco-2/PBMC 100 μg/ml, and PBMC 1 μg/ml, the non-toxic concentrations (Fig. S1). A detailed description of the procedure is in Supplementary Materials.

### Lactate dehydrogenase assay

Cytotoxicity of LPS treatments was evaluated by lactate dehydrogenase (LDH) activity in cell culture medium using the Cytotoxicity Detection Kit PLUS (Roche, USA) as described previously (Binó et al. 2016).

### TEER

Trans-epithelial electric resistance (TEER) measures changes in the permeability of the monolayer which increases during inflammation. TEER of the Caco-2 monolayer was measured by ECIS^®^ TEER24 (Applied BioPhysics, USA) at two time points: before the assembly of the co-culture and after 4 days of the exposure to LPS. For the measurement at the end of the experiment, the co-culture was disassembled and only inserts with Caco-2 cell monolayer were put in the TEER measuring plate. As a negative control, two empty inserts with a cultivation medium were used.

### Wound healing assay

Wound healing assay studies changes in cell migration and proliferation under pro-inflammatory conditions. Confluent HaCaT cells grown on the 96-well plate were used for the assay. Half of the plates was treated with mitomycin at a final concentration of 1 μg/ml for 2 h to stop the proliferation and observe only migration. After PBS wash, the wounds were made by BioTek AutoScratch Wound Making Tool (Agilent, USA), the cells were washed again, supplemented with HaCaT culture medium, and treated with LPS as mentioned above. The cells were maintained in BioTek BioSpa 8 Automated Incubator (Agilent) and photos of the wounds were acquired by BioTek Cytation 5 Imaging Reader (Agilent) each 6 h for 24 h. The area of the wounds was calculated in the Gen5 program (Agilent).

### Flow cytometry

Under pro-inflammatory conditions, the monocytes differentiate into macrophages and change the expression of specific surface molecules CD16 and CD14. After 4 days of exposure, PBMC were collected and the expression of the markers was measured by flow cytometry. Detailed procedure is provided in the Supplementary Materials. The data were analyzed in the FlowJo program (BD Life Sciences, USA). The gating strategy is described in Fig. S2. tSNE analysis included four parameters (FSC, SSC, CD14, CD16).

### ELISA

Upon pro-inflammatory stimuli, cells produce cytokines to drive inflammation and regeneration. Concentrations of selected cytokines were measured in the media by commercially available ELISA kits according to the manufacturer’s instructions.

### Western blot

Pro-inflammatory activation can change the expression of various proteins by the cell. To study these changes, the cells were lysed, sonicated (Sonopuls, Bandelin, Germany), and boiled (10 min). Protein concentration was measured by BCA assay (Pierce^TM^ BCA ProteinAssay kit, ThermoFisher Scientific, USA) according to the manufacturer’s instructions, the samples were adjusted to the same concentration and supplemented with 5 × Laemli buffer. The SDS-PAGE electrophoresis and western blotting were done as described previously (Pekarova et al. 2015). The detailed procedures can be found in Supplementary Materials.

### Statistical analysis

Data are presented as a mean ± SD. The number of independently repeated experiments (*n*) is stated in the figure legend. Statistical analysis was performed using GraphPad Prism version 9.5.1 for Windows (GraphPad Software, USA). Outliers were identified by the ROUT method and filtered out. Significant difference from negative control (named as “control” throughout the text and figures) was determined by unpaired *t*-test. In case of disagreement of variances, the Mann–Whitney test was used. Data normalized to control were analyzed by one-sample *t*-test. *p < 0.05, **p < 0.01.

## Results

### Taxonomy evaluation, endotoxin activity of CyanoHAB-LPS, and smooth vs. rough LPS composition

Cyanobacteria accounted for at least 47% biovolume of the phytoplankton biomass (Table S1 and Fig. S3). The exception was the sample B, where picocyanobacterium *Aphanocapsa holstatica* constituted ~ 50% of the cell count, but its share in the biovolume was < 0.1% due to its small cell size compared to the larger taxa. The LPS yield (Table S3) significantly positively correlated with cyanobacterial biovolume in the biomass, in contrast to the eukaryotic algae biovolume, while the correlation with the endotoxin activity of the biomass was weaker and not significant (Fig. S4). There were only weak and nonsignificant correlations between LPS yield and qPCR-based estimate of cyanobacterial and bacterial biomass in the sample, while the endotoxin activity did not correlate with any of the parameters (Fig. S5). Endotoxin activity of the majority of LPS samples fell within the range of tens of EU/mg LPS (Table S3). The highest activity was shown by sample G with almost 8 000 EU/mg LPS. Three other samples (A, K, and N) reached more than 1 000 EU/mg. In contrast, samples B and O exhibited relatively low endotoxin activity with less than 10 EU/mg LPS. However, even the highest endotoxin activity was not comparable to *E. coli* LPS with more than 3.5 × 10^6^ EU/mg LPS. The migration patterns of isolated LPS in SDS-PAGE showed variation among individual samples (Fig. S6). Pro-Q Emerald 300 staining exhibited higher sensitivity for CyanoHAB-LPS than silver staining, although certain samples (e.g., H and N) exhibited only faint staining even with Pro-Q (Fig. S6). Common characteristics among cyanoHAB-LPS included dominant presence of smooth LPS with larger molecular weight (bands > 20 kDa), while rough or semi-rough LPS observed in *E. coli* LPS (bands < 15 kDa) were absent, except the samples O, S and J (Pro-Q only). Contamination with protein or DNA (Fig. S7 or S8) was not detected, while RNA contamination in the isolated CyanoHAB-LPS was below < 2% w/w (Fig. S8).

### Biomass DNA composition highly differs

**Fig. 1:**
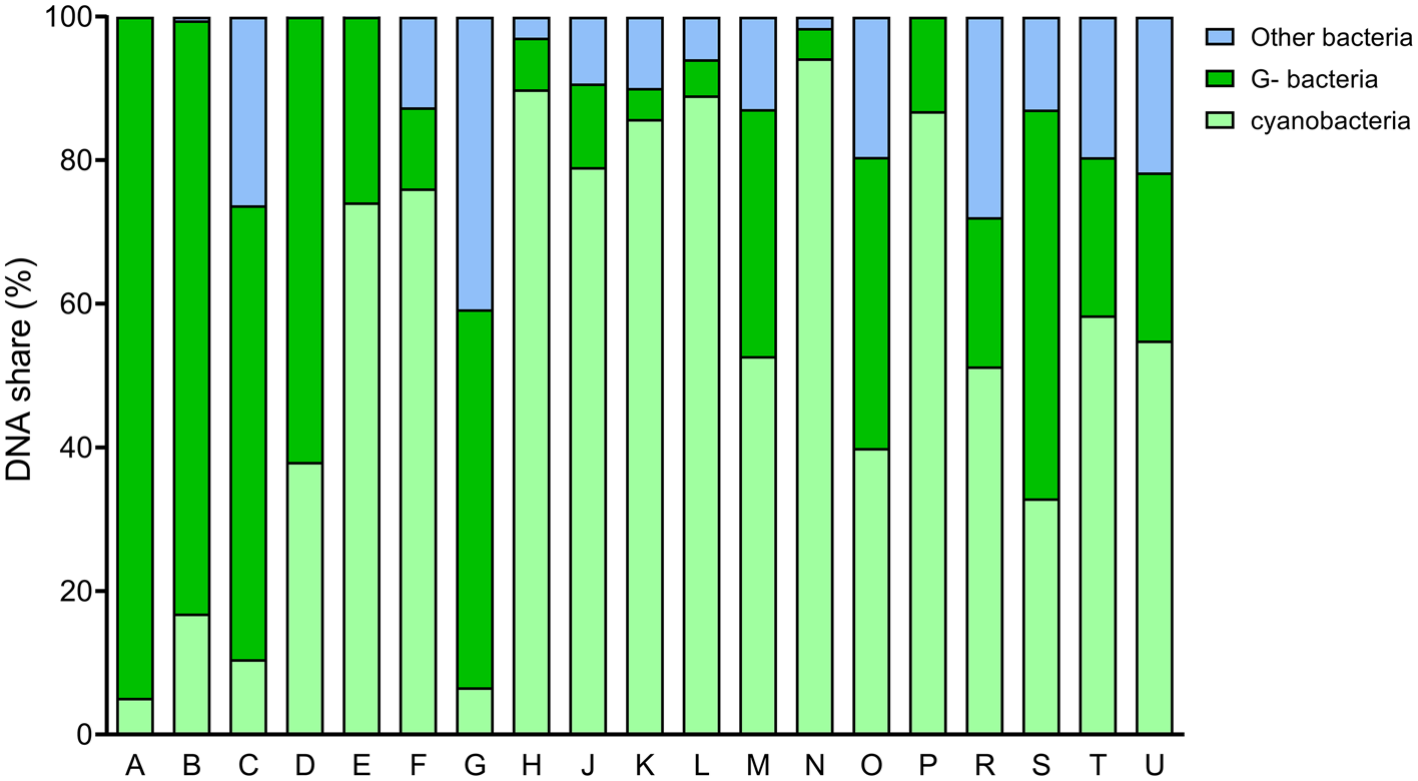
Quantification of DNA sequences in CyanoHAB biomasses using qPCR. TaqMan Probes specifically targeting total heterotrophic bacteria, Gram-negative bacteria (G-), and cyanobacteria were employed to quantify their respective DNA shares. The results are presented as a percentage of cyanobacteria, G- and the remaining bacterial population (i.e., total heterotrophic bacteria excluding G-) among the total quantified DNA

qPCR analysis showed four samples (A, D, E, and P) to contain only DNA of G- bacteria and cyanobacteria (Fig. 1). Seven samples (N, H, L, P, K, J, and F) showed cyanobacterial DNA share higher than 75% (94.2%, 89.98%, 89.0%, 86.8%, 85.7%, 79.0%, 76.1%, resp.), other five samples were in the range 50–75% (E = 74.1%, T = 58.3%, U = 54.8%, M = 52.7%, and R = 51.3%). In contrast, two biomasses contained less than 10% of cyanobacterial DNA (G = 6.6% and A = 5.1%). Similarly, the share of G- bacteria DNA varied among the samples with six biomasses above 50% (A = 94.9%, B = 82.6%, C = 63.2%, D = 62.0%, S = 54.1%, and G = 52.6%) and four biomasses under 10% (H = 7.2%, L = 5.1%, K = 4.3%, and N = 4.2%).

### Three CyanoHAB-LPS induced pro-inflammatory cytokine production in keratinocytes

**Fig. 2:**
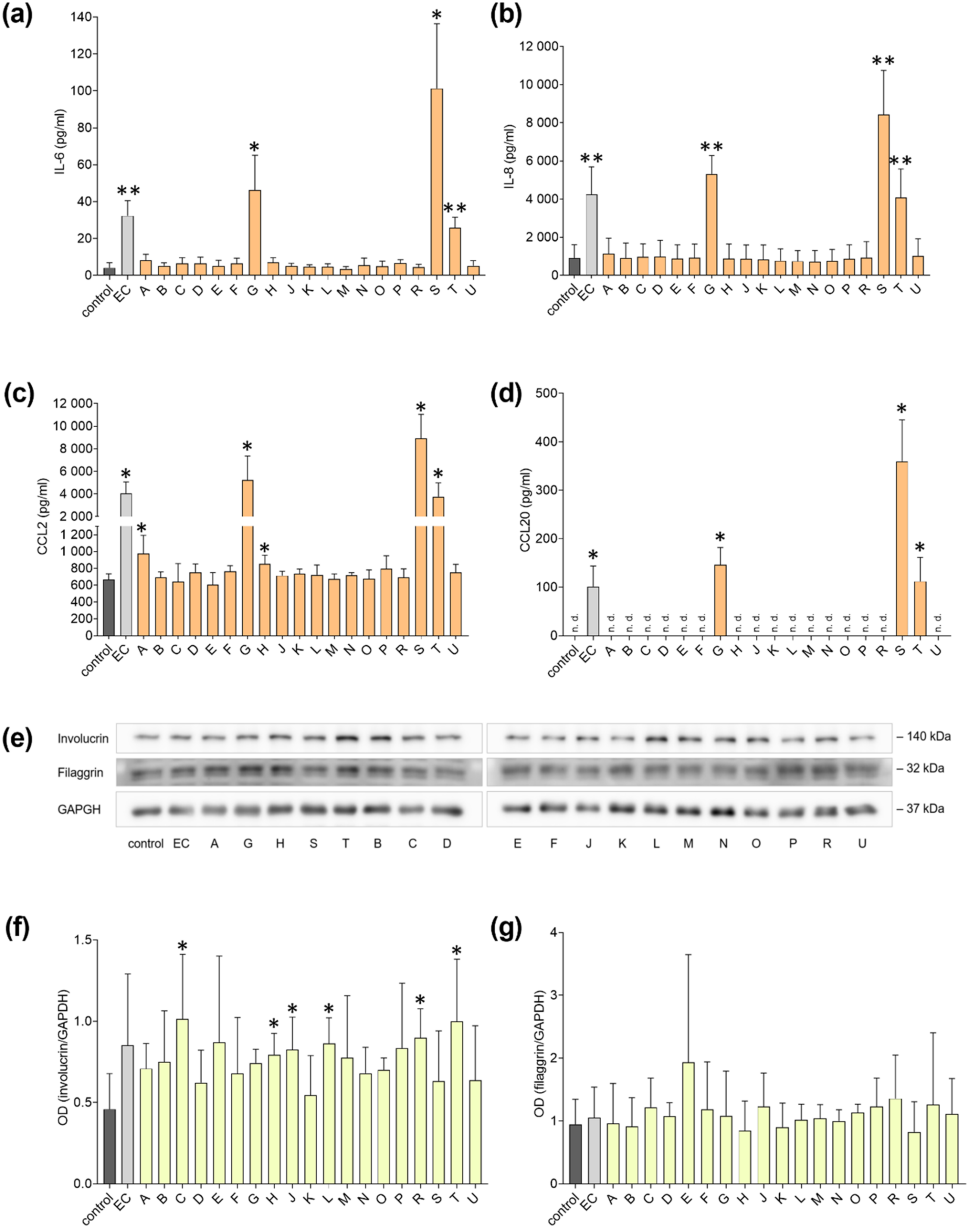
Response of keratinocytes to LPS exposure. Confluent HaCaT cells were treated with 19 CyanoHAB-LPS from 18 recreational water bodies (A–U) at a final concentration of 50 μg/ml for 1 day. Negative control was untreated, and LPS from *E. coli* (EC) was used as a positive control. The concentration of **a** IL-6, **b** IL-8, **c** CCL2, and **d** CCL20 in the culture medium was determined by ELISA. Protein expression of involucrin and filaggrin by the cells: **e** representative blots and relative concentration of **f** involucrin and **g** filaggrin expressed as the optical density of the bands. Data are expressed as the mean ± SD. Data were statistically analyzed by unpaired *t*-test. *n* = 4. *p < 0.05, **p < 0.01

Three LPS samples (G, S, and T) induced substantial elevation of all four detected pro-inflammatory cytokines (Fig. 2a–d). The other two samples (A and H) caused just a slight increase in CCL2 production. The cytokine response after exposure to the remaining samples was comparable to the negative control. Although filaggrin and involucrin expression decreases during inflammation, we did not observe any down-regulation of these proteins (Fig. 2e–g). On the contrary, the level of involucrin was elevated upon exposure to some otherwise inactive samples (C, H, J, and L) and even to one cytokine release-inducing LPS (T). In the wound healing assay, no significant effects on HaCaT cell migration and proliferation were observed (Fig. S9).

### One CyanoHAB-LPS induced pro-inflammatory response in the intestinal model

**Fig. 3:**
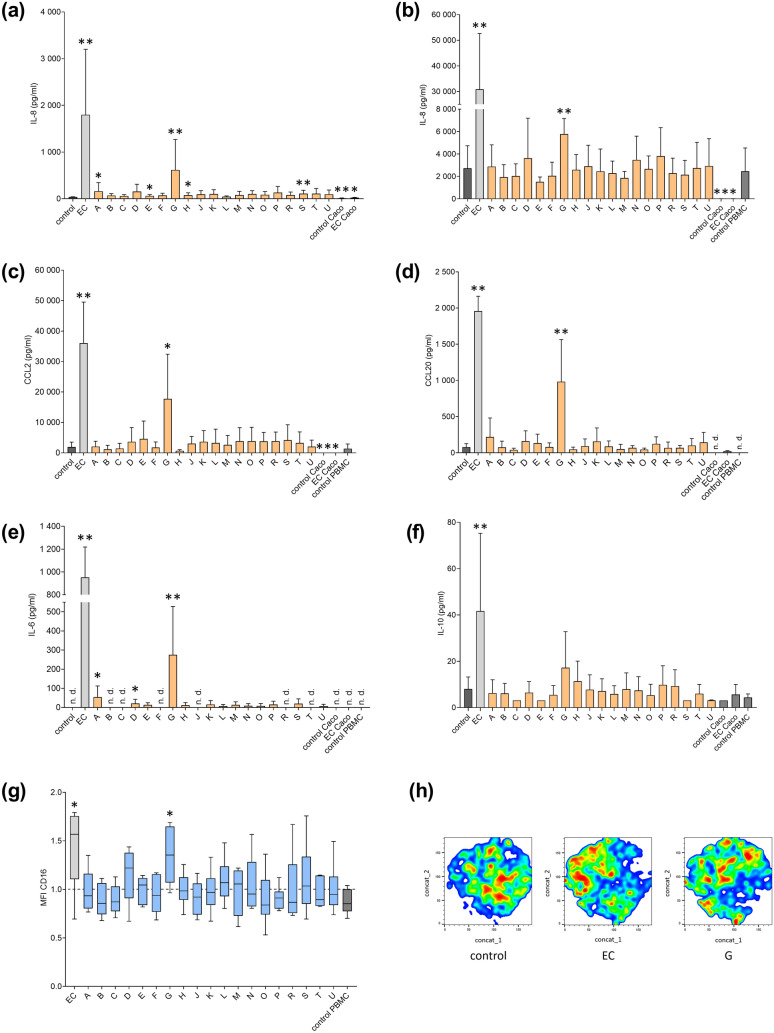
Response of Caco-2/PBMC co-culture to LPS exposure. Co-culture of differentiated Caco-2 cells and human PBMC were treated with 19 CyanoHAB-LPS from 18 recreational water bodies (A–U) at a final concentration of 100 μg/ml for 4 days. Negative control was untreated, and LPS from *E. coli* (EC) was used as a positive control. Single cultures of differentiated Caco-2 cells or PBMC were used as controls of the model. The concentration of **a** IL-8 in the apical medium and **b** IL-8, **c** CCL2, **d** CCL20, **e** IL-6, and **f** IL-10 in the basolateral medium was determined by ELISA (*n* = 8). **g** Expression of CD16 by monocytes in PBMC fraction was determined by flow cytometry (*n* = 6, dashed line represents control). **h** Representative tSNE visualization of monocytes from samples with significantly increased CD16 expression. Data are expressed as the mean ± SD. Data were statistically analyzed by unpaired *t*-test (**a**–**f**) or one sample *t*-test (**g**). *p < 0.05, **p < 0.01. *MFI* median fluorescence index

Only one LPS sample (G) was highly active throughout most of the measured parameters, inducing the production of all pro-inflammatory cytokines and expression of CD16 (Fig. 3a–g). The tSNE analysis confirmed the results showing similar clustering of the cells in sample G compared to the positive control, indicating changes in cell size and expression of markers typical for activated monocytes/macrophages (Fig. 3h). Few other samples (A, D, E, H, and S) elevated level of one or two cytokines but never to the same extent as sample G. Despite the pro-inflammatory responses to several CyanoHAB-LPS evident from cytokine levels and monocyte activation, the integrity of the Caco-2 monolayer did not appear to be compromised. No drop in TEER or change in the tight junction protein expression was observed (Fig. S10).

### Almost all CyanoHAB-LPS induce some pro-inflammatory response in PBMC

**Fig. 4:**
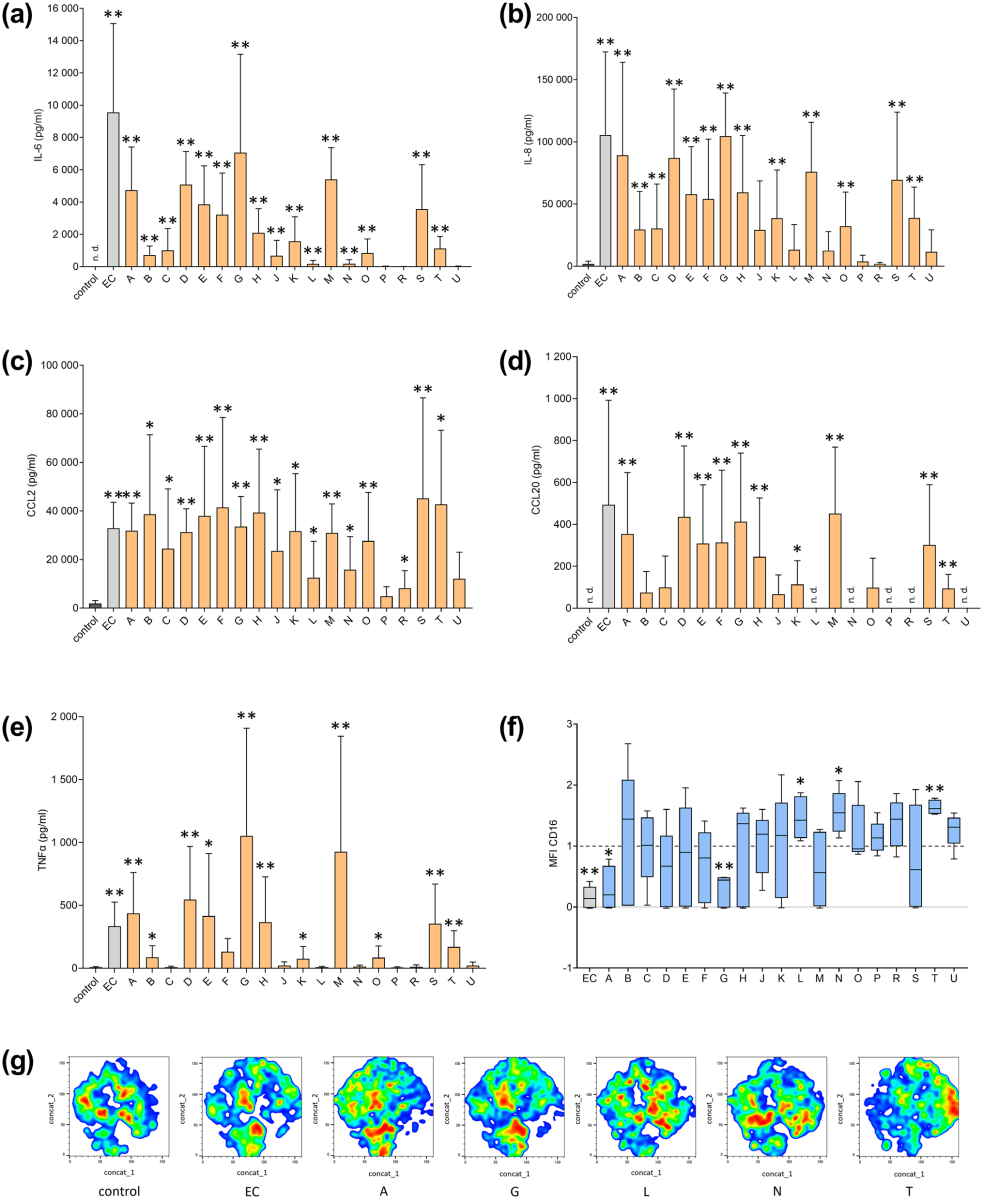
Response of PBMC to LPS exposure. Human PBMC were treated with 19 CyanoHAB-LPS from 18 recreational water bodies (A–U) at a final concentration of 1 μg/ml for 4 days. Negative control was untreated, and LPS from *E. coli* (EC) was used as a positive control. The concentration of **a** IL-6, **b** IL-8, **c** CCL2, **d** CCL20, and **e** TNFα in the culture medium was determined by ELISA (*n* = 6). **f** Expression of CD16 by monocytes in PBMC fraction was determined by flow cytometry (*n* = 5, dashed line represents control). **g** Representative tSNE visualization of monocytes from samples with significantly changed CD16 expression. Data are expressed as the mean ± SD. Data were statistically analyzed by unpaired *t*-test (**a**–**e**) or one sample *t*-test (**f**).*p < 0.05, **p < 0.01. *MFI* median fluorescence index

The PBMC response dynamics were completely different from that of epithelial cells. Most LPS samples caused an elevation of one cytokine level at least (Fig. 4a–e). Some samples significantly changed all studied parameters (e.g., A and G), while some significantly influenced few and seemed to be inactive in others (e.g., C and L). Three LPS samples (P, R, and U) caused no or very weak pro-inflammatory response. CD16 expression patterns differed from the results of the co-culture model (Fig. 4f). Sample G and *E. coli* LPS caused a decrease in CD16 expression which was opposite to the effect of indirect PBMC activation. Sample A worked similarly. On the other hand, three other samples (L, N, and T) elevated CD16 expression. Samples L and N acted similarly enhancing some cytokine production to a comparable extent, while sample T significantly increased the levels of all measured cytokines. In accordance with this observation, clustering of the cell subpopulations was similar in samples G and A together with the positive control on the one side and L with N on the other side (Fig. 4g). Clustering of sample T cells was quite unique among the PBMC-activating LPS.

## Discussion

In this study, 19 CyanoHAB biomasses from various recreational water bodies in the Czech Republic were collected during two consecutive seasons to investigate the bioactivity of their LPS. To study this issue, we used three different in vitro cell models and a commercial endotoxin activity assay. Among the individual samples, only sample G exhibited pro-inflammatory responses in all three in vitro models, and its response was often comparable to *E. coli* LPS. Sample G also had the highest endotoxin activity from all studied CyanoHAB-LPS. Similar patterns of effects in PBMC responses were observed in sample A but its pro-inflammatory effect on epithelial models was weaker. The dominant cyanobacteria in sample A were *Planktothrix agardhii*, accompanied by *Aphanizomenon* sp., and *Planktolyngbya* sp., while sample G was dominated by *Dolichospermum flos-aquae*, *Sphaerospermopsis* sp., and *Aphanizomenon issatchenkoi*. Our previous results show CyanoHAB-LPS isolated from *Dolichospermum flos-aquae*-dominated water bloom to be more bioactive than the ones isolated from *Planktothrix agardhii*- and *Aphanizomenon flos-aquae*-dominated blooms (Raptová et al. 2023; Skočková et al. 2023). Other samples with outstanding profile of effects in our CyanoHAB-LPS collection were samples S and T. They were (together with sample G) highly active in HaCaT keratinocytes. The more bioactive sample S was dominated by cyanobacteria *Planktothrix agardhii*, *Raphidiopsis* (ex. *Cylindrospermopsis*) *raciborskii*, *Microcystis* sp., and *Pseudanabaena* sp., sample T by *Dolichospermum* sp., *Aphanizomenon klebahnii*, and *Woronichinia* sp. The other samples dominated by *Planktothrix agardhii* (H, N) or *Dolichospermum* sp. (E) showed much lower bioactivity in the epithelial cell models. The only exception was the induction of CD16 expression in PBMC by sample N, although its other effects on these cells were either much lower than those of others or were not even detectable. With these results it seems that dominance of cyanobacteria *Dolichospermum* or *Planktothrix* might serve as some kind of a predictor of potential biological activity of the LPS produced by the water bloom but for sure it does not stand alone.

*Microcystis* is one of the most common bloom forming cyanobacterial genera in the fresh waters around the world (Huisman et al. 2018). Samples dominated by this genus showed no pro-inflammatory effects in epithelial cells, in contrast to our other previous results, where one particular *Microcystis*-dominated CyanoHAB-LPS induced strong pro-inflammatory effects in HaCaT as well as Caco-2 cells (Raptová et al. 2023; Skočková et al. 2023). In PBMC, the effect differed. Samples L (*Microcystis*) and N (dominated with *Planktothrix* but more than 20% of biomass made up of *Microcystis*) showed similar pattern in cytokine activation and above all significant induction of CD16 expression and similar clustering of PBMC subpopulations. The other *Microcystis*-dominated samples (F, J, K, and M) activated the PBMC to produce cytokines but they did not change their differentiation status. Finally, samples P and U were completely inactive. Previously, *Microcystis aeruginosa*-dominated CyanoHAB-LPS were shown to activate human whole blood cells as well as isolated neutrophils (Bláhová et al. 2013; Moosová et al. 2019) but our results show that not all the environmental mixtures have this ability.

The other LPS-producing organisms in the water blooms are G- bacteria. Their share highly varied among the samples from approximately 4% (sample N) to 94% (sample A). Samples with the highest in vitro bioactivity had G- bacteria share from low (T = 22%) to average (G = 53%, S = 54%) and high (A = 95%). The share of G- bacteria also was not reflected by the endotoxin activity of the CyanoHAB-LPS, where samples S and T showed less than one hundred EU/mg LPS, sample A displayed 1 227 EU/mg LPS, and sample G exhibited almost eight thousand EU/mg LPS. Our analysis also showed that endotoxin activity of the CyanoHAB-LPS did not significantly correlate with quantities of G- bacteria, cyanobacteria or other bacteria in the biomass. None of the above mentioned parameters could clearly eludcidate the bioactivity of the CyanoHAB-LPS.

No relationships between endotoxin activity, in vitro activity and SDS-PAGE migration pattern were observed. In general, CyanoHAB-LPS samples were dominated by bands > 20 kDa, indicating dominance of smooth LPS, which is in agreement with previous studies (Fujii et al. 2012; Raptová et al. 2023; Skočková et al. 2023; Swanson-Mungerson et al. 2019). In contrast to *E. coli* LPS, CyanoHAB-LPS were stained more intensively with Pro-Q Emerald 300 compared to silver staining, which suggests differences in their chemistry, such as presence of more hydrophobic and poorly charged LPS that are preferentially stained with Pro-Q Emerald 300 (Snyder et al. 2009), or presence of acidic sugars for which silver staining has a low affinity (Fujii et al. 2012). Very faint LPS staining was observed for samples H and N dominated by *Planktothrix agardhii*, which corresponds to other reports with LPS from CyanoHABs or cultures of *Planktothrix* (Raptová et al. 2023; Skočková et al. 2023).

Overall, our results demonstrate that certain environmental LPS associated with CyanoHABs can indeed induce pro-inflammatory effects in human skin and intestinal epithelial cells, as well as in immune cells in vitro. Although a majority of the tested CyanoHAB-LPS in our study did not exhibit significant effects in the epithelial cells, about 15–20% of samples were found to be active in at least one cell model. Moreover, these effects were induced in vitro at endotoxin concentrations that can be found in natural waters with CyanoHABs. The literature reports significant variability in the endotoxin activity of CyanoHAB-LPS, with values ranging from 1500 times lower to 5000 times higher than those observed for the most active sample G in our study (Bernardová et al. 2008; Bláhová et al. 2013; Moosová et al. 2019; Skočková et al. 2023). However, the potency of individual LPS effects and response patterns across the different in vitro models and endpoints varied significantly in this study, mostly regardless of endotoxin activity. The main driver behind these differences remains to be identified. So far, the observed activities did not exhibit a clear correlation with the taxonomic composition of the phytoplankton community, the relative share of microbial groups in the biomass, endotoxin activity of the LPS, or LPS migration pattern or band intensity in the SDS-PAGE. The resulting effects of CyanoHAB-LPS likely depend on the specific composition and abundance of various LPS structures in the complex environmental sample and their mixture interactions with cellular receptors. Predicting these effects from other parameters is challenging. Further research is needed to better understand the complexities involved in CyanoHAB-LPS effects.

### Supplementary Information

Below is the link to the electronic supplementary material.

Supplementary file1 (PDF 1577 KB)

## Data Availability

The datasets generated during and/or analyzed during the current study are available from the corresponding author on a reasonable request.
