# Supplementary material for: Cyanobacterial harmful bloom lipopolysaccharides: pro-inflammatory effects on epithelial and immune cells in vitro
Source: Arch Toxicol. 2023 Dec 8;98(2):481–91. doi: 10.1007/s00204-023-03644-8 (PMC10794361; doi:10.1007/s00204-023-03644-8)
Supplement: Supplementary file 1 — Supplementary file1 (PDF 1577 KB) [file 204_2023_3644_MOESM1_ESM.pdf]

Cyanobacterial harmful bloom lipopolysaccharides: pro-inflammatory effects on epithelial and immune cells *in vitro*

Skočková V.[1,2], Raptová P.[1,2], Pospíchalová K.[1], Sovadinová I.[3], Sychrová E.[3], Smutná, M.[3], Hilscherová, K.[3], Babica P.[3,4], Šindlerová L.[1*]

[1]Department of Biophysics of Immune System, Institute of Biophysics of the Czech Academy of Sciences, Brno 61200, Czech Republic; skockova@ibp.cz (V.S.); raptova@ibp.cz (P.R.); katerina.pospichalova@ibp.cz (K.P.)
[2]Department of Experimental Biology, Faculty of Science, Masaryk University, Brno 62500, Czech Republic
[3]RECETOX, Faculty of Science, Masaryk University, Brno 62500, Czech Republic; sovadinova@recetox.muni.cz (I.S.); eliska.sychrova@recetox.muni.cz (E.S.); marie.smutna@recetox.muni.cz (M.S.); klara.hilscherova@recetox.muni.cz (K.H.); pavel.babica@recetox.muni.cz (P.B.)
[4]Department of Experimental Phycology and Ecotoxicology, Institute of Botany of the Czech Academy of Sciences, Brno 60200, Czech Republic
[*]Correspondence: sindler@ibp.cz (L.Š.)

Tab. S1 Sampling localities, dates and dominants of CyanoHABs used for LPS isolation.

Sample code	Locality	GPS	Sampling date	Dominant groups and species (based on %biovolume)
A	Dvorský rybník Pond	48°51'32.960"N, 17°4'21.948"E	Jun 19, 2019	***Planktothrix agardhii*** (26.1%), ***Planktolyngbya limnetica*** (10.5%), ***Aphanizomenon* sp.** (7.4%), algae (52.7%)
B	Horní štítarský rybník Pond	48°55'50.545"N, 15°51'4.550"E	Jul 10, 2019	***Dolichospermum flos-aquae*** (0.5%), ***Chroococcus limneticus*** (0.2%), ***Aphanocapsa holstatica*** (<0.1%), algae (99.3%)
C	Hejtman Pond	48°57'52.352"N, 14°56'28.401"E	Aug 07, 2019	***Woronichinia naegeliana*** (82.3%), ***Microcystis wesenbergii*** (10.4%), algae (1.9%)
D	Starý Kanclíř Pond	48°58'18.773"N, 14°53'55.219"E	Aug 07, 2019	***Sphaerospermopsis aphanizomenoides*** (26%), ***Aphanizomenon klebahnii*** (19.2%), ***Microcystis* sp.** (16.1%) [mainly *M. aeruginosa* (10.4%)], ***Raphidiopsis raciborskii*** (8.7%), ***Dolichospermum* sp.** (6.1%) [mainly *D. crassum* (5.8%)], ***Planktolyngbya* sp.** (6%), algae (16.4%)
E	Výtopa Pond	48°59'3.797"N, 14°52'47.859"E	Aug 07, 2019	***Dolichospermum* sp.** (95.6%) [mainly *D. planctonicum* (38.4%), *D. crassum* (23.7%), *D. ucrainicum* (20.9%), *D. flos-aquae* (10.5%)], algae (0.2%)
F	Nový Kanclíř Pond	48°57'50.984"N, 14°53'50.893"E	Aug 07, 2019	***Microcystis* sp.** (91.7%) [mainly *M. flos-aquae* (45.8%), *M. viridis* (23%), *M. aeruginosa* (10.7%), *M. wesenbergii* (8.7%)], algae (0.8%)
G	Ruda Pond	48°56'51.739"N, 14°46'24.711"E	Aug 07, 2019	***Dolichospermum flos-aquae*** (43.2%), ***Sphaerospermopsis* sp.** (16.1%) [mainly *S. aphanizomenoides* (9.9%), *S. reniforme* (6.2%)], ***Aphanizomenon issatchenkoi*** (16.1%), algae (21.2%)
H	Kamenský Šutrák Pond	48°50'47.773"N, 16°48'34.583"E	Aug 27, 2019	***Planktothrix agardhii*** (90.2%), algae (1.5%)
J	Františkův rybník Pond	48°43'5.907"N, 16°51'48.166"E	Aug 27, 2019	***Microcystis aeruginosa*** (94.8%), algae (<0.1%)
K	Nové Mlýny – střed Reservoir	48°53'43.067"N, 16°36'57.501"E	Aug 27, 2019	***Microcystis* sp.** (96.9%) [mainly *M. botrys* (80.4%), *M. aeruginosa* (16.5%),] algae n.d.
L	Vranov Reservoir, Bítov bay	48°56'13.533"N, 15°43'25.236"E	Aug 27, 2019	***Microcystis* sp.** (97.5%) [mainly *M. flos-aquae* (88.1%), *M. aeruginosa* (7.7%)], algae (0.5%)
M	Letovice Reservoir	49°33'25.999"N, 16°32'12.920"E	Sep 17, 2019	***Microcystis* sp.** (99.8%), [mainly *M. flos-aquae* (42.2%), *M. viridis* (33.5%), *M. wesenbergii* (20.3%)], algae n.d.
N	Olšovec Pond	49°20'11.679"N, 16°45'36.605"E	Sep 17, 2019	***Planktothrix agardhii*** (68.5%), ***Microcystis novacekii*** (22.5%), ***Aphanizomenon* sp.** (6%), algae (3%)
O	Mlýnský rybník Pond	48°47'6.200"N, 16°49'18.614"E	Sep 17, 2019	***Raphidiopsis raciborskii*** (72.2%), algae (25.7%)
P	Brno Reservoir	49°14'20.086"N, 16°30'31.262"E	Sep 24, 2019	***Microcystis* sp.** (69.2%) [mainly *M. aeruginosa* (45.7%), *M. novacekii* (20.8%)], ***Woronichinia naegeliana*** (25.4%), algae (2%)
R	Letovice Reservoir	49°33'13.753"N, 16°33'20.420"E	Aug 27, 2020	***Microcystis* sp.** (65.5%) [mainly *M. aeruginosa* (49.3%), *M. viridis* (16.1%)], ***Woronichinia naegeliana*** (34.5%), algae n.d.
S	Konventský rybník Pond	49°34'49.600"N, 15°56'17.721"E	Aug 27, 2020	***Planktothrix agardhii*** (22.8%), ***Raphidiopsis raciborskii*** (8%), ***Microcystis* sp.** (7%), ***Pseudanabaena* sp.** (7.1%), algae (43.1%)
T	Kachlička Pond	49°33'8.816"N, 15°25'57.236"E	Aug 27, 2020	***Dolichospermum* sp.** (30.6%) [mainly *D. crassum* (23.6%)], ***Aphanizomenon klebahnii*** (15.9%), ***Woronichinia* sp.** (5.8%), algae (40.2%)
U	Trnávka Reservoir	49°31'22.777"N, 15°13'1.081"E	Aug 27, 2020	***Microcystis* sp.** (76%) [mainly *M. flos-aquae* (57.7%), *M. aeruginosa* (10.2%), *M. viridis* (7%)], ***Aphanizomenon klebahnii*** (14.8%), algae (6.8%)

Cyanobacterial genera and/or species with more than 5% biovolume share in the concentrated biomass are listed. The only exception is the sample B, where all the cyanobacterial species are listed due to a very low biovolume share of cyanobacteria in this mixture. The sum of biovolume share of all eukaryotic algae is also provided. n.d.= not detected.

Tab. S2 List of specific primers and FAM-BHQ1-labeled probes used for Taqman qPCR

Target	Primer Sets and Probe Sequences (5′->3′)
Cyanobacteria	Forward: ACGGGTGAGTAACRCGTRA
	Reverse: CCATGGCGGAAAATTCCCC
	Probe: CTCAGTCCCAGTGTGGCTGNTC
Total heterotrophic bacteria	Forward: TCCTACGGGAGGCAGCAGT
	Reverse: GGACTACCAGGGTATCTAATCCTGTT
	Probe: CGTATTACCGCGGCTGCTGGCAC-3′
Gram-negative bacteria	Forward: AACTGGAGGAAGGTGGGGAT
	Reverse: AGGAGGTGATCCAACCGCA
	Probe: GACGTAAGGGCCATGAGGACTTGACGTC

Primers and probes are based on the publication: Lang-Yona, N.; Lehahn, Y.; Herut, B.; Burshtein, N.; Rudich, Y. Marine aerosol as a possible source for endotoxins in coastal areas. Sci. Total. Environ. 2014, 499, 311–318. https://doi.org/10.1016/j.scitotenv.2014.08.054.

Tab. S3 LPS yield from the CyanoHAB biomasses and endotoxin activity. The endotoxin activity of the isolated CyanoHAB-LPS and the water from the site of the collection was assessed by Pyrogene^{TM} assay. The endotoxin activity of the final concentration used for the treatment in the *in vitro* experiments was calculated. The green color denotes biomasses, blue water *in situ*, and yellow culture media.

Sample code	LPS yield (mg LPS/g biomass d.w.)	PyroGene^{TM} (EU/mg LPS)	PyroGene^{TM} (EU/g biomass d.w.)	PyroGene^{TM} (EU/ml water)	Caco-2/PBMC (EU/ml medium)	HaCaT (EU/ml medium)	PBMC (EU/ml medium)
A	6.2	1 226.8	7 619	884	122.68	61.34	1.23
B	5.1	5.2	27	901	0.52	0.26	0.01
C	49.6	17.4	865	368	1.74	0.87	0.02
D	23.6	20.3	478	647	2.03	1.02	0.02
E	66.9	30.1	2 011	935	3.01	1.51	0.03
F	20.5	35.9	735	174	3.59	1.80	0.04
G	11.4	7 870.7	89 726	317	787.07	393.54	7.87
H	3.3	12.3	41	898	1.23	0.62	0.01
J	23.1	435.0	10 047	623	43.50	21.75	0.44
K	8.3	1 864.0	15 546	51	186.40	93.20	1.86
L	20.1	45.5	913	147	4.55	2.28	0.05
M	33.9	81.2	2 754	7 438	8.12	4.06	0.08
N	13.7	1 617.7	22 195	135	161.77	80.89	1.62
O	9.5	7.9	74	230	0.79	0.40	0.01
P	15.2	170.4	2 586	44	17.04	8.52	0.17
R	47.0	38.4	1 807	695	3.84	1.92	0.04
S	20.0	70.8	1 416	141	7.08	3.54	0.07
T	24.0	21.6	518	164	2.16	1.08	0.02
U	47.0	73.9	3 473	51	7.39	3.70	0.07

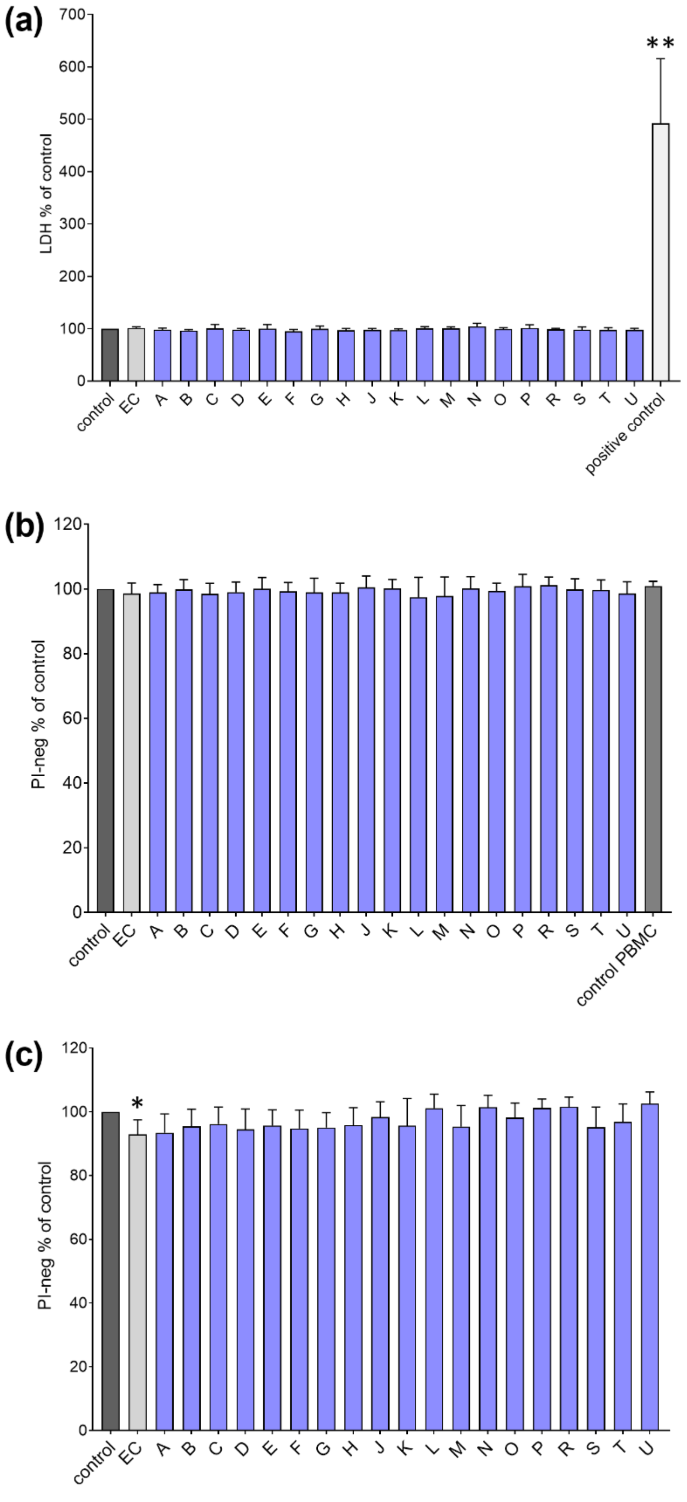

Fig. S1 Cytotoxicity of used LPS concentrations. (a) The concentration of LDH in culture medium after treatment of HaCaT cells with LPS in concentration 50 µg/ml for 1 day, n = 3. Cells lysed by supplier-provided lysis buffer were used as a positive control. (b) Percentage of viable (PI-negative) PBMC after treatment of Caco-2/PBMC co-culture with LPS in concentration 100 µg/ml for 4 days, n = 6. (c) Percentage of viable (PI-negative) cells after treatment of PBMC with LPS in concentration 1 µg/ml for 4 days, n = 5. Data were converted to a percentage of the control (untreated cells) and expressed as the mean ± SD. Data were statistically analyzed by one-sample *t*-test. * $p < 0.05$

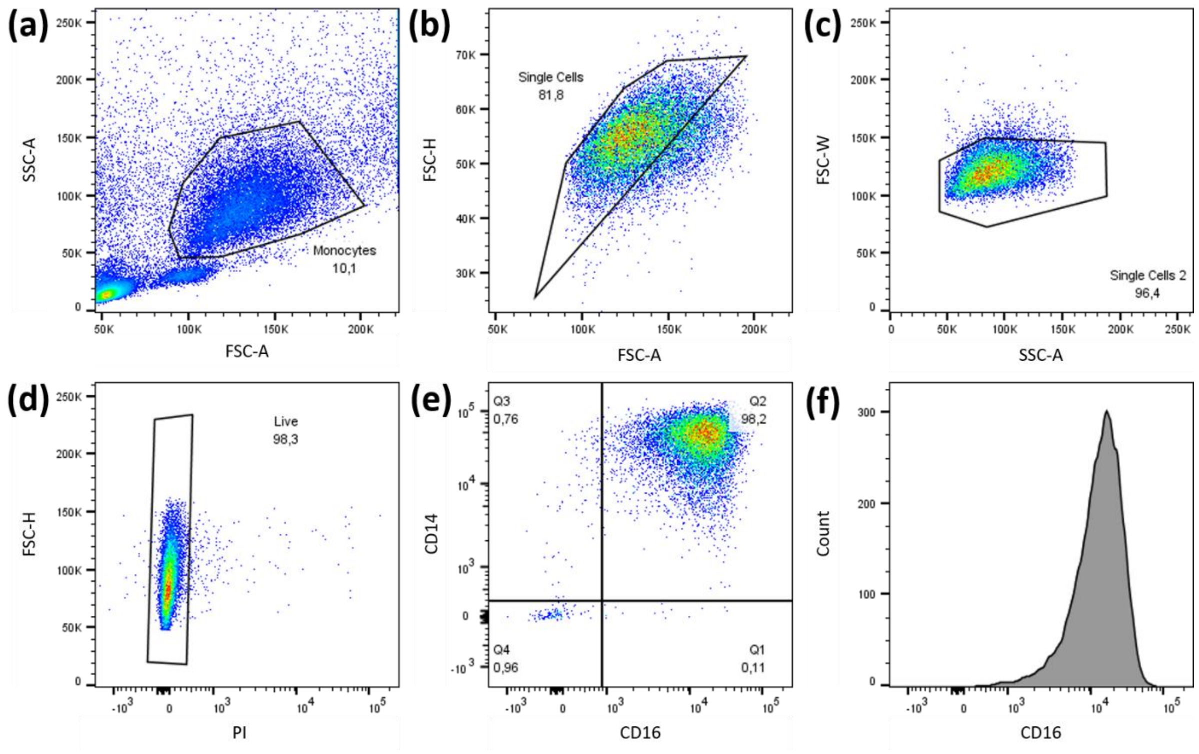

Fig. S2 Flow cytometry gating strategy. (a) Monocytes, based on FSC-A/SSC-A. (b) Single cells, based on FSC-A/FSC-H. (c) Single cells, based on SSC-A/SSC-W. (d) Live cells, based on propidium iodide negativity. (e) Determination of CD14/CD16 double negative cells (quartile Q4). (f) CD16 expression in quartiles Q1 – Q3.

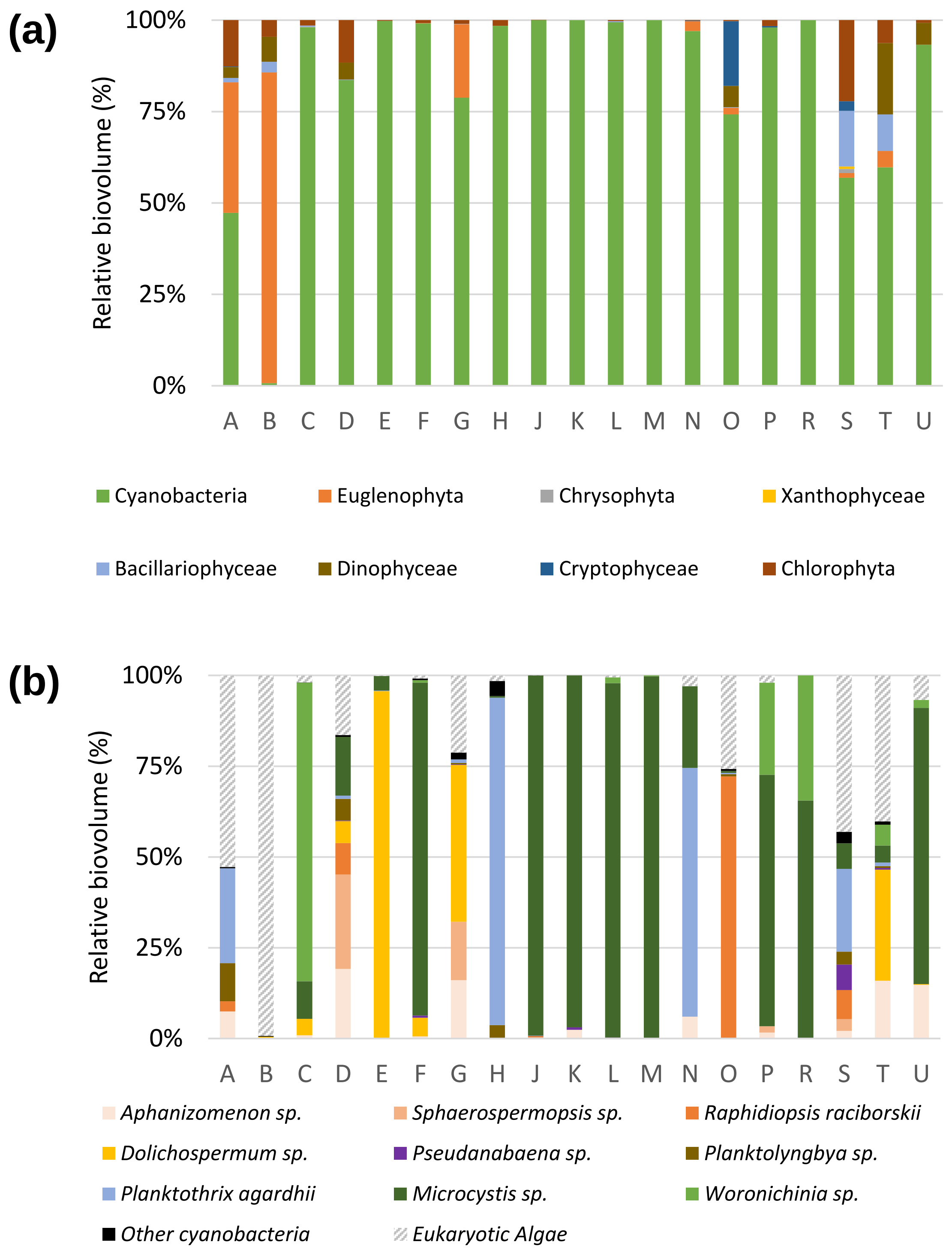

Fig. S3 Composition of CyanoHABs used for LPS isolation as a percentage of biovolume. (a) Biovolume share of the main phytoplankton groups. (b) Biovolume of dominant cyanobacterial genera or species.

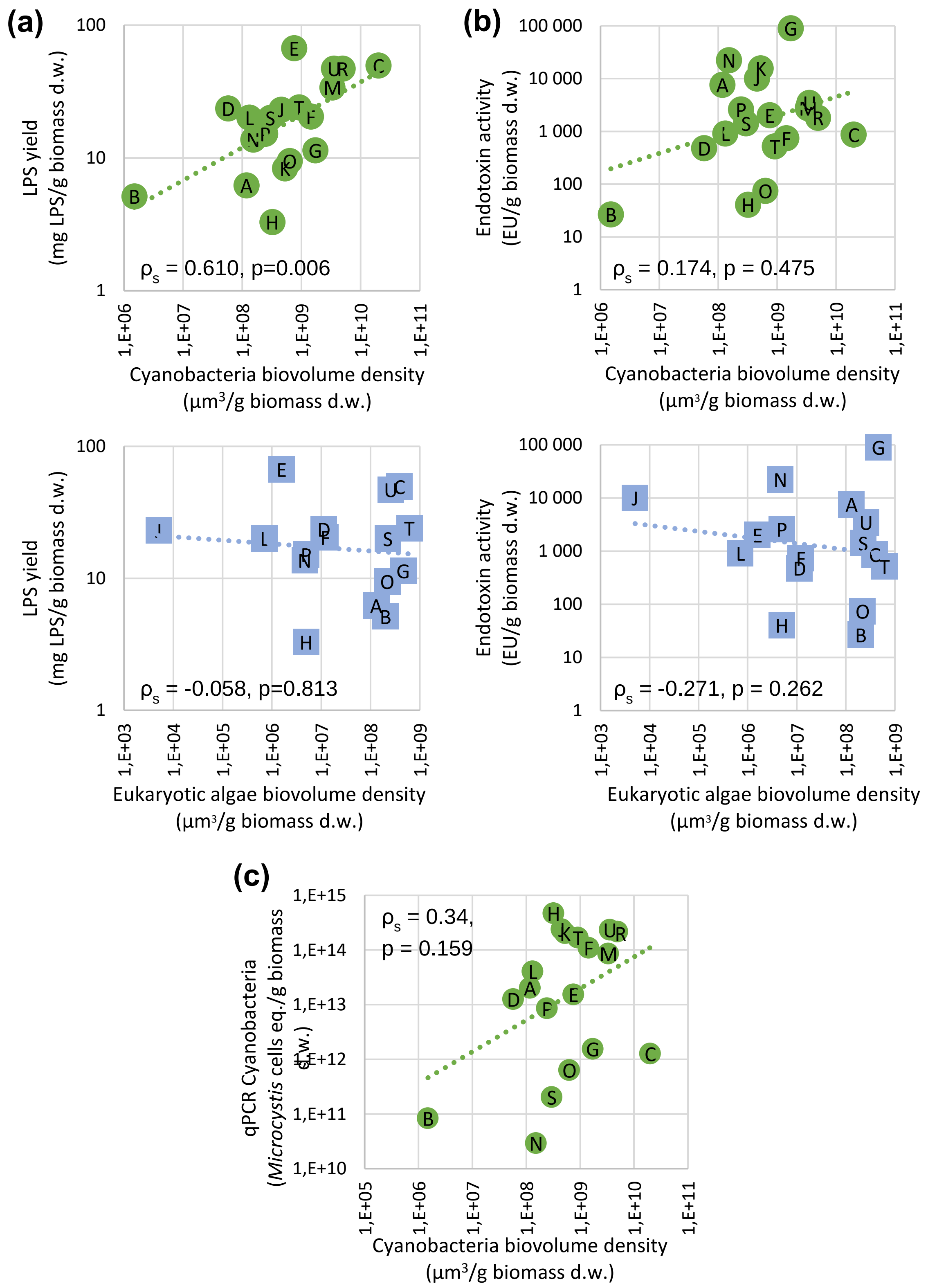

Fig. S4 Relationship between phytoplankton biovolume and LPS yield or endotoxin activity in CyanoHABs. (a) Cyanobacteria or (b) eukaryotic algae biovolumes normalized per dry weight (d.w.) of biomass and plotted against LPS yield (left) or endotoxin activity (right) per CyanoHAB biomass d.w. (c) Biovolume of cyanobacteria compared to qPCR-based cyanobacterial cell count estimates (equivalents of *Microcystis*) per CyanoHAB biomass d.w. Letters (A-U) represent the sample codes, ρ_s = Spearman's rank correlation coefficient and two-tailed *p* value

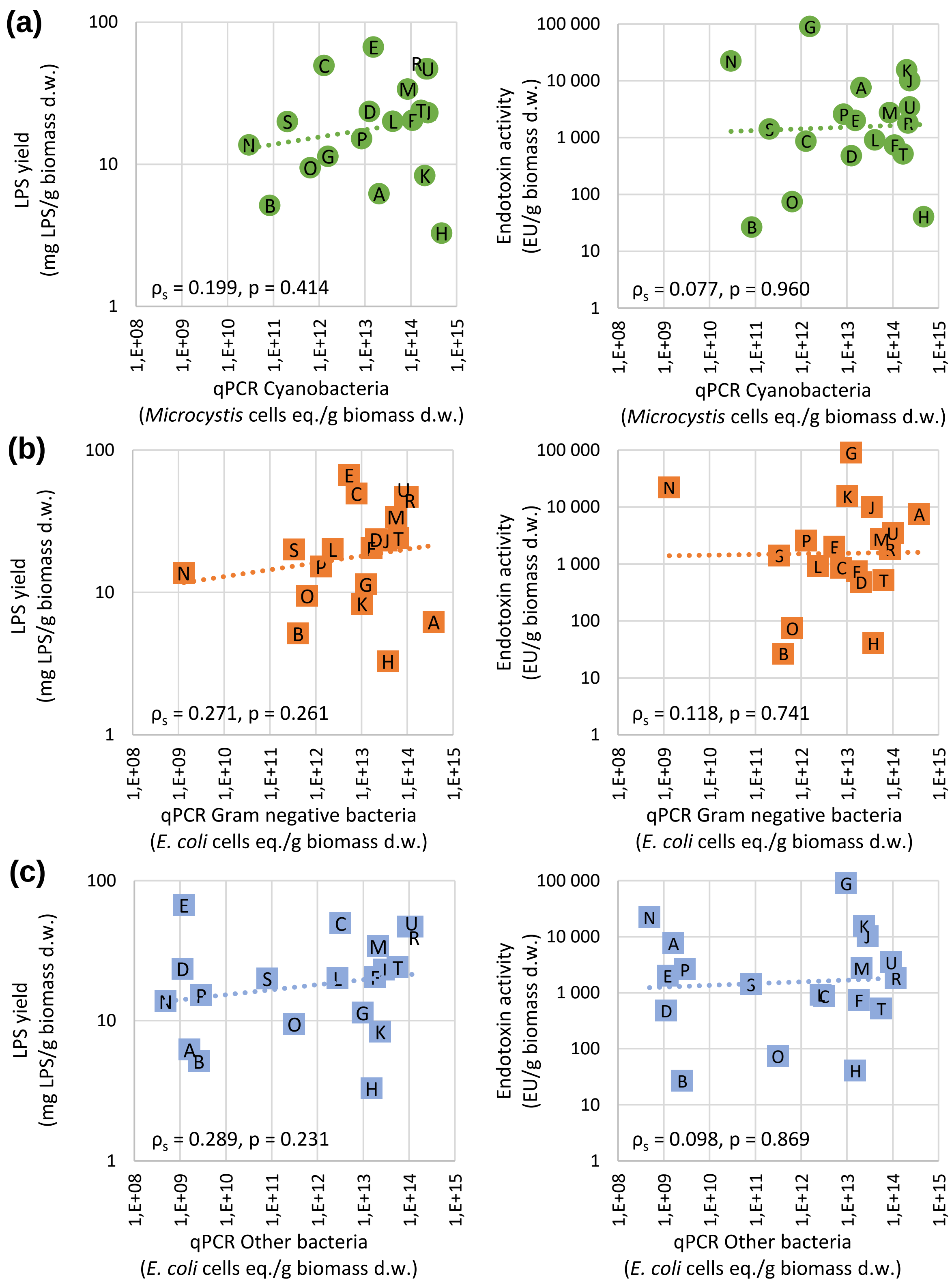

Fig. S5 Relationship between qPCR-based estimates of microorganism quantity of microorganisms in CyanoHABs and LPS yield or endotoxin activity. (a) Cyanobacteria, (b) Gram-negative bacterial, and (c) other bacterial (total bacteria without Gram-negative) cell counts (equivalents of *Microcystis* or *E. coli*) as estimated by qPCR per CyanoHAB biomass dry weight (d.w.), and plotted against either LPS yield (left) or endotoxin activity (right) per CyanoHAB biomass d.w. Letters (A-U) represent the sample codes, ρ_s= Spearman's rank correlation coefficient, and two-tailed *p*-value.

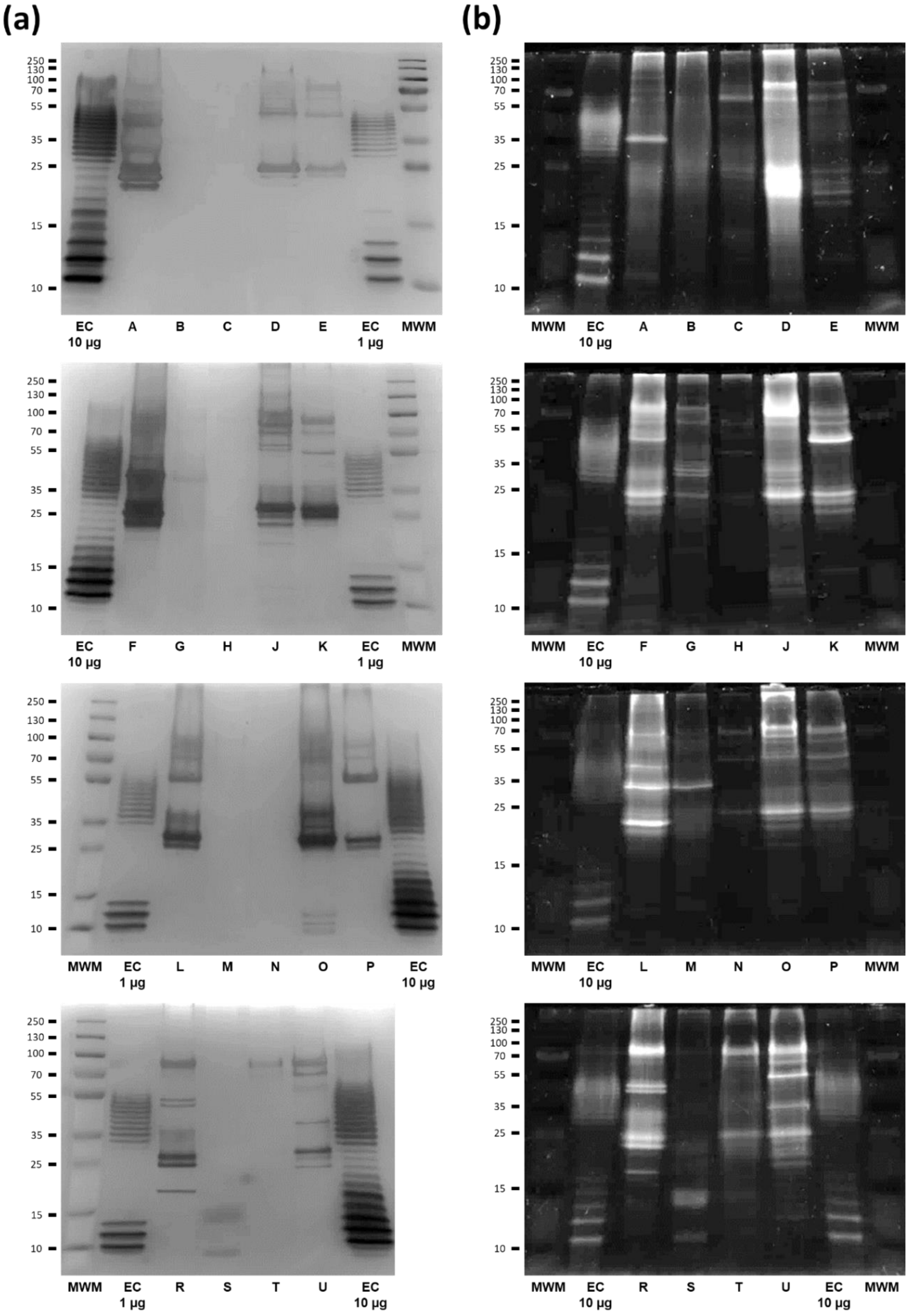

Fig. S6 Separation of LPS by SDS-PAGE followed with (a) silver or (b) Pro-Q™ Emerald 300 staining. LPS isolated from CyanoHAB biomasses (samples A–U) were loaded at 10 µg per lane. EC – purified LPS from *E. coli* O111:B4 (Sigma-Aldrich L2630), MWM – molecular weight marker (PageRuler™ Plus Prestained Protein Ladder, Thermo 26619).

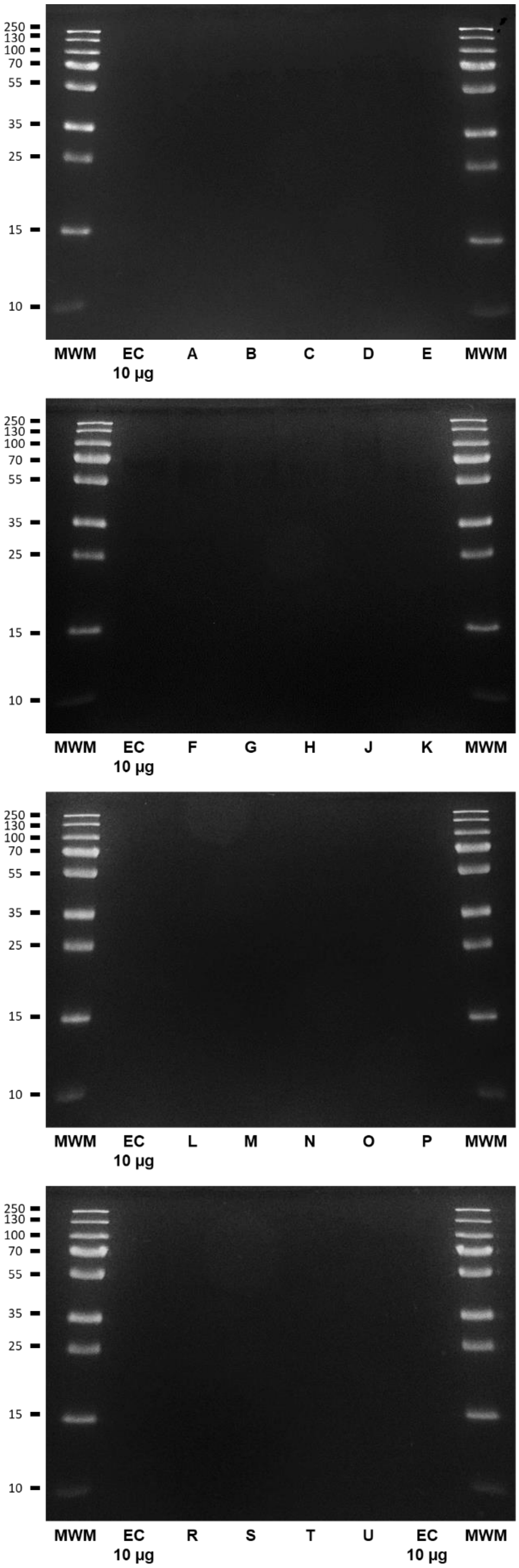

Fig. S7 Separation of LPS by SDS-PAGE followed with SYPRO™ Ruby Protein staining. LPS isolated from CyanoHAB biomasses (samples A–U) were loaded at 10 µg per lane. EC – purified LPS from *E. coli* O111:B4 (Sigma-Aldrich L2630), MWM – molecular weight marker (PageRuler™ Plus Prestained Protein Ladder, Thermo 26619).

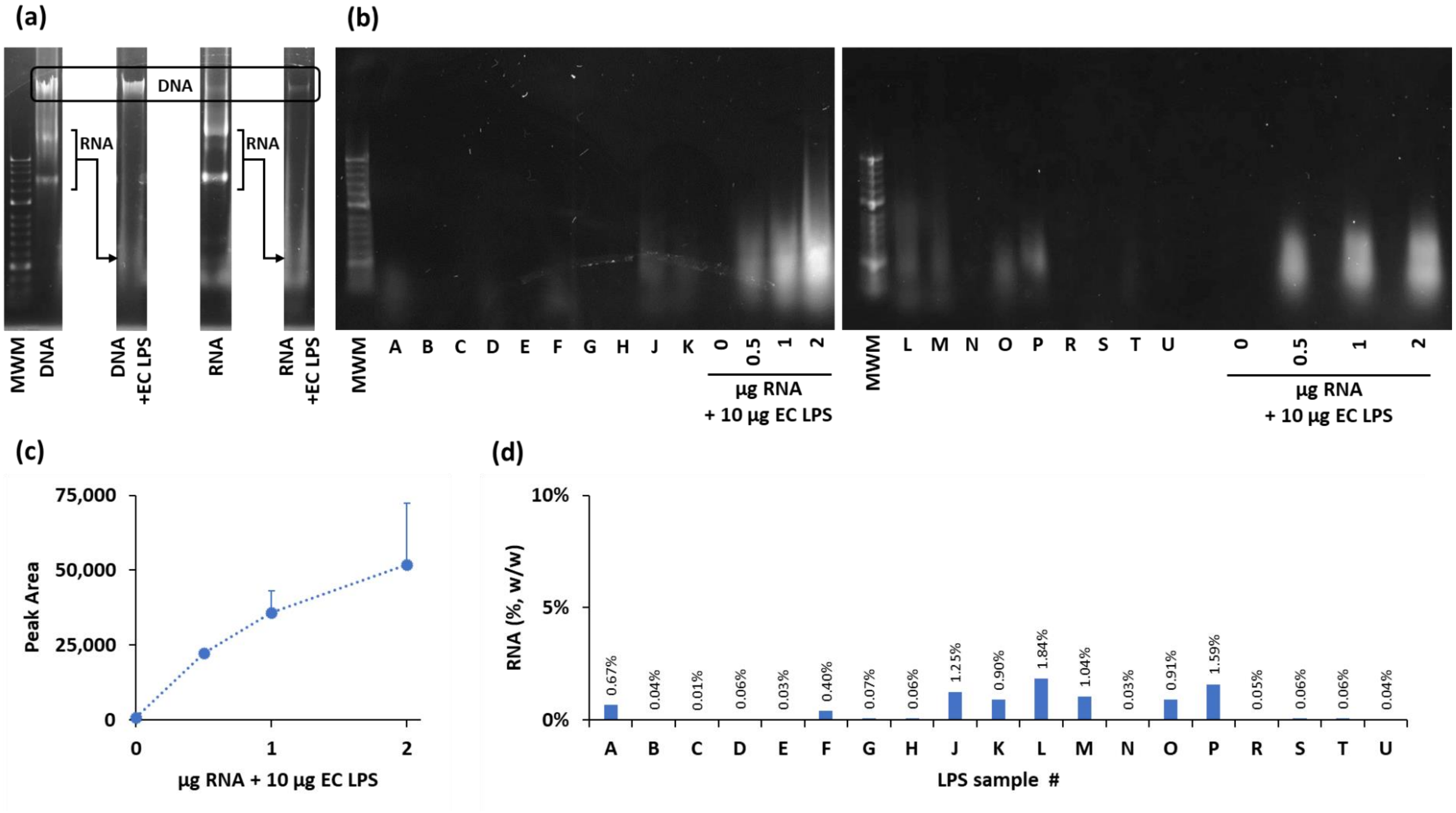

Fig. S8 Agarose gel electrophoresis of LPS and nucleic acids followed by ethidium bromide staining. (a) Model DNA or RNA (0.5 µg) was loaded alone or in the mix with 10 µg LPS from *E. coli* O111:B4 (EC LPS). The presence of LPS was found to affect the migration pattern of RNA bands and cause their smearing. (b) Detection of nucleic acids in the LPS samples isolated from CyanoHABs (samples A–U) loaded at 10 µg per lane. As a control, 10 µg LPS from *E. coli* O111:B4 (EC LPS) was mixed with 0–2 µg of semipurified RNA. (c) Densitometric evaluation of 0–2 µg RNA + 10 µg *E. coli* O111:B4 (EC LPS). (d) Estimated amounts of RNA in the LPS samples isolated from CyanoHABs (samples A–U) based on densitometric analysis of the agarose gels, expressed as % of sample weight (w/w). Model DNA and RNA were isolated from a fish cell line NKA-1.

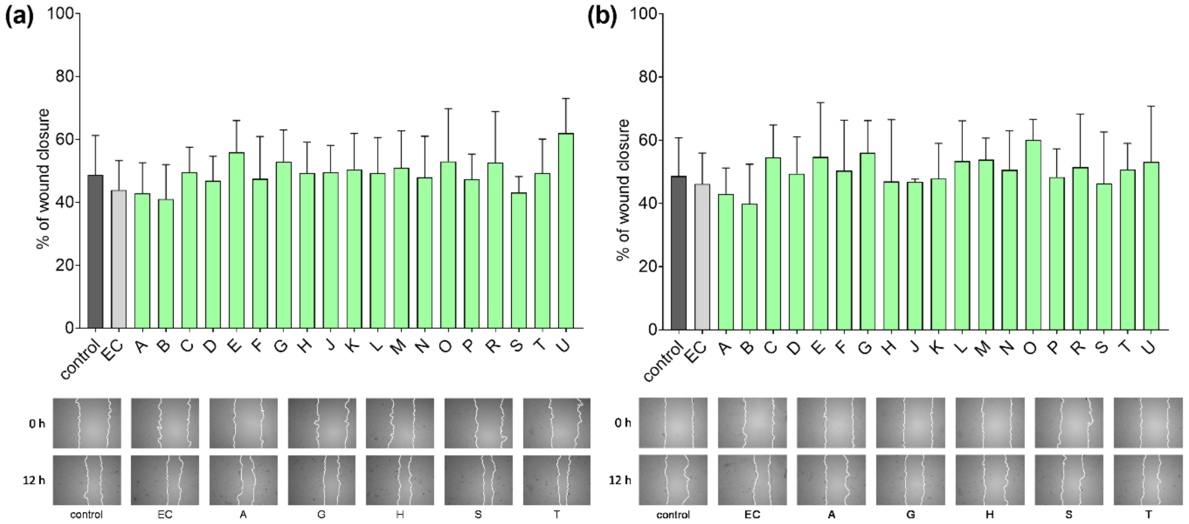

Fig. S9 Migration potential of keratinocytes. Confluent HaCaT cells were treated with 19 CyanoHAB-LPS from 18 recreational water bodies (A–U) at a final concentration of 50 µg/ml for 4 days. Negative control was untreated, and LPS from *E. coli* (EC) was used as a positive control. Wound closure of the scratched monolayer was assessed after 12 h. Cell proliferation was either (a) unaffected or (b) stopped by mitomycin c pre-treatment (1 µg/ml, 2 h). (c) Selection of representative images. Data are expressed as the mean ± SD. Data were statistically analyzed by unpaired *t*-test.

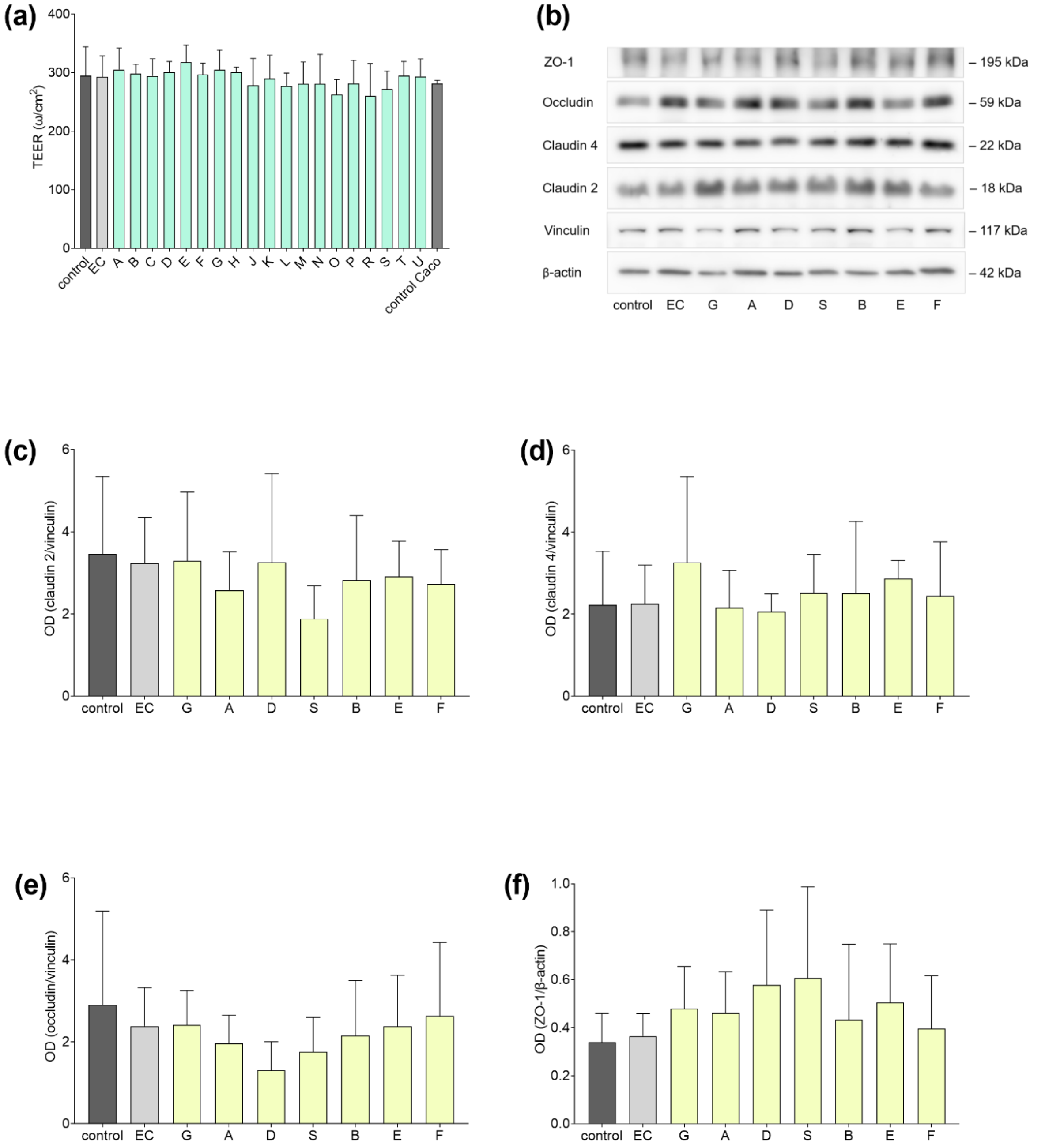

Fig. S10 Caco-2 monolayer integrity. Co-culture of differentiated Caco-2 cells and human PBMC were treated with 19 CyanoHAB-LPS from 18 recreational water bodies (A–U) at a final concentration of 100 μg/ml for 4 days. Negative control was untreated, and LPS from *E. coli* (EC) was used as a positive control. Simple cultures of differentiated Caco-2 cells were used as a control of the model (control Caco). (a) Trans-epithelial electric resistance (TEER) of the Caco-2 monolayer, n = 4. Expression of tight junction proteins: (b) representative blots and relative concentration of (c) claudin 2, (d) claudin 4, (e) occludin, and (f) zonula occludens 1 expressed as the optical density of the bands, n = 4 – 8. Data are expressed as the mean ± SD. Data were statistically analyzed by unpaired *t*-test.

DETAILED METHODOLOGY

LPS isolation

1 g of lyophilized cyanobacterial biomass was mixed with 50 ml of MilliQ water and sonicated using an ultrasonic bath (10 min). The suspension was then heated to 68 °C, mixed with 50 ml of pre-warmed 90% phenol, and stirred for 20 min at 68 °C. After cooling to 4 °C, the mixture was centrifuged (5 630 g, 30 min, 4 °C), and the supernatant (aqueous phase with LPS) was collected. The phenol layer was re-extracted with 50 ml of MilliQ water, and pooled supernatants were purified by dialysis (48 h) using cellulose membranes (33x21 mm, Sigma-Aldrich, USA, D9652) against MilliQ water (1 l) containing toluene (10 µl/l) to prevent bacterial contamination. After 24 h of dialysis, the water was changed and dialysis continued for another 24 h. The dialyzed extracts were centrifuged (5 630 g, 30 min, 4 °C), collected as supernatants and lyophilized. The resulting semi-purified freeze-dried extract of LPS was resuspended in 3.75 ml of 0.1M Tris-HCl buffer (pH 7.4) containing 25 µg/ml ribonuclease A (RNase, Sigma-Aldrich, R4642) and incubated for 16 h at 37 °C. Next, 3.75 ml of 90% phenol in 0.1M Tris-HCl was added, vortexed, and incubated for 4 min at room temperature (RT). The solution was centrifuged (18 410 g, 15 min, RT), the aqueous phase was separated and purified for 48 h by dialysis for a second time (see above) and then lyophilized. The purified LPS (freeze-dried powder) was weighed to assess the content of LPS in the biomass and kept at -20 °C. Isolated LPS were dissolved (at a concentration of 10 mg/ml) in MilliQ water for characterization by SDS-PAGE or agarose electrophoresis, or in PBS with 0.1% BSA (v/v, Sigma-Aldrich) for PyroGene™ rFC or *in vitro* cell assays.

SDS-PAGE and agarose electrophoresis of CyanoHAB-LPS

Isolated LPS samples (10 µg) were diluted with MilliQ water and 4× Laemmli loading buffer (Bio-Rad, Hercules, CA, USA, 161-0747) containing 2-mercaptoethanol (final concentration 357 mM), and heated for 10 min at 65 °C. Commercially available LPS purified from *Escherichia coli* serotype O111:B4 (Sigma-Aldrich, L2630) was used as a positive control (1 or 10 µg per lane). The 4% (w/v) acrylamide stacking gels were used for SDS-PAGE in combination with 10–20% (w/v) gradient separating gels (1 mm thickness) prepared from 40% (w/v) acrylamide/bis-acrylamide solution, 37.5:1 (Serva, Heidelberg, Germany) using a Mini-Protean gel casting and electrophoresis system (Bio-Rad). Electrophoresis was conducted at 130 V, and the gels were then washed with deionized water, and stacking gels were removed.

For the silver staining of LPS, the washed gels were fixed with 40% (v/v) ethanol-5% (v/v) acetic acid overnight at RT. Then, the fixed gels were oxidized with 0.7% (w/v) periodic acid in 40% (v/v) ethanol-5% (v/v) acetic acid for 5 min, and washed with deionized water (2×5 min and 1×10 min). The silver staining solution was freshly prepared by mixing 28 ml of 0.1M sodium hydroxide, 2.3 ml of 24% (w/w) ammonium hydroxide, 5 ml of 20% (w/v) silver nitrate (Sigma-Aldrich) and filled up to 150 ml with deionized water. The gels were incubated in the staining solution for 10 min and then washed with deionized water (2×5 min and 1×10 min). The developing process was carried out with 50 µg/ml citric acid in 0.05% (v/v) formalin for approximately 2-3 min, followed by washing with deionized water to achieve suitable levels of background staining. Finally, the gels were fixed with 5% (v/v) acetic acid for 1 min, washed, and documented under white light (Alliance Q9 Advanced system, Uvitec Cambridge, UK).

LPS were stained with the Pro-Q™ Emerald 300 Lipopolysaccharide Gel Stain Kit (ThermoFisher Scientific, Waltham, MA, USA) according to the manufacturer's protocol. Washed gels were fixed with 50% (v/v) methanol-5% (v/v) acetic acid for 45 min at RT and then washed with 3% (v/v) acetic acid for 2×15 min. LPS were oxidized for 30 min with a periodic acid-based oxidizing solution supplied with the kit. The gels were washed again with 3% (v/v) acetic acid (3×15 min) and then incubated for 90 min with 1× Pro-Q™ Emerald 300 staining buffer. Before UV-light visualization and documentation (Alliance Q9 Advanced), the gels were washed with 3% acetic acid for 2×20 min. To visualize protein contamination of LPS, the gels were restained overnight with SYPRO® Ruby (ThermoFisher Scientific), washed with 10% methanol with 7% acetic acid (v/v) for 30 min and finally with deionized water for 2×5 min, prior documentation (Alliance Q9 Advanced).

Agarose gel electrophoresis of LPS and nucleic acids was conducted as reported previously (Sychrová et al., 2022, https://doi.org/10.1016/j.etap.2022.103869), using 1% gel, TAE buffer, and Bioline HyperLadder™ 50 bp molecular weight marker (MWM). The gels were stained using a 1% solution of ethidium bromide and documented with the Alliance Q9 Advanced system (Cambridge, UK). LPS from *Escherichia coli* serotype O111:B4 (Sigma-Aldrich) and its mixture with DNA or RNA from a fish cell line NKA-1 (isolated as reported previously in Součková et al., 2023, https://doi.org/10.1016/j.aquatox.2023.106517) were used as positive controls and to create

a calibration curve for the RNA amount in the mixture with LPS. Densitometric analysis was conducted using ImageJ software (Schneider et al., 2012, https://doi.org/10.1038/nmeth.2089).

DNA extraction

Briefly, freeze-dried biomass (4–10 mg) was resuspended in 0.5 ml of 0.15 M NaCl/0.1 M EDTA solution and homogenized using three freeze-thaw cycles with liquid nitrogen. Samples were then centrifuged (7 200 g, 10 min), the supernatant was discarded, and the pellet was resuspended in 0.5 ml of TE buffer. Then, 1 µl of RNase (10 mg/ml) was added and the samples were incubated at 37 °C for 1 h. Afterwards, 100 µl of lysozyme (50 mg/ml) was added, and the samples were incubated at 37 °C for 30 min. After incubation, 10 µl of protein kinase K (50 mg/ml) and 2% (final concentration) sodium dodecyl sulfate (SDS) were added, and samples were incubated at 55 °C for 1 h. Then, selective precipitation was performed by adding 150 µl of 5M NaCl to the tubes followed by 0.1 volumes (of total volume) of 10% cetyltrimethylammonium bromide stock solution, mixed by inversion, and incubated at 65 °C for 10 min. For purification (to allow for protein precipitation), 1 volume of chloroform was added, and tubes were incubated on ice for 30 min. Then, samples were centrifuged (7 200 g, 10 min, 4 °C), and the supernatant was transferred to fresh tubes, mixed with 0.6 volumes of isopropanol, and incubated overnight at 4 °C. Following this, samples were centrifuged (16 000 g, 30 min, 4 °C), the isopropanol supernatant was discarded, and the pellet was washed twice with 1 ml of 70% ethanol. Finally, samples were centrifuged (16 000 g, 30 min, 4 °C), the supernatant discarded, the pellet air-dried, and then resuspended in 30 µl of TE buffer. The concentration and quality of the extracted DNA were checked via NanoDrop (Thermo Scientific) and agarose gel electrophoresis. The isolated DNA underwent further processing through qPCR.

Cell lines media

Caco-2 medium: high-glucose Dulbecco's Modified Eagle's Medium (DMEM; Gibco) supplemented with 10% heat-inactivated low endotoxin fetal bovine serum (FBS; v/v, PAA), 1% non-essential amino acids (v/v, Gibco), 1mM sodium pyruvate (Gibco), 2mM L-glutamine (Gibco), and 100 U/ml of penicillin and 100 µg/ml of streptomycin (Pen/Strep; Gibco)

HaCaT medium: high-glucose DMEM supplemented with 10% FBS, 2 mM L-glutamine, and Pen/Strep

PBMC isolation

30 ml of blood was collected to 300 µl of 0.5M EDTA. 6ml aliquots of blood were centrifuged (400 g, 25 min, 20 °C, the lowest acceleration, no brake; Rotina 420, Hettich Zentrifugen, Germany), buffy coat was collected, pooled into 3 tubes, and washed twice with 8 ml of PBS+1mM EDTA (150 g, 10 min, 20 °C, the lowest acceleration, no brake). The washed buffy coat was diluted by 1mM EDTA/PBS up to 8 ml. Each 2 ml of suspension was carefully layered on 2 ml of Histopaque 1077 and centrifuged (400 g, 30 min, 18 °C, the lowest acceleration, no brake). PBMC layers were collected, pooled, and washed with 5 ml of 1mM EDTA/PBS (500 g, 10 min, 20 °C). Washed PBMC were resuspended in PBS and counted (CASY Cell Counter, Innovatis). PBMC were diluted in RPMI (Gibco) with 20% FBS up to concentration 2×10^6 cells/ml, seeded in 24 plates, 500 µl per well, and placed in the incubator. After 2 h, cells were extremely gently washed with pre-warmed PBS and 800 µl of fresh RPMI with 20% FBS was added. Cells prepared in this way were used either for the assembly of a co-culture or for direct LPS treatment.

Treatment

Confluent HaCaT cells were treated with LPS at a final concentration of 50 µg/ml. Positive control was treated with *E. coli* O111:B4 LPS (Sigma-Aldrich) diluted in 0.1% BSA in PBS, while the negative control cells received no treatment. After 1 day of exposure, the medium was collected, and the cells were washed with ice-cold PBS and prepared for western blot.

Co-culture was assembled by putting an insert with differentiated Caco-2 cells into the well with PBMC. LPS was applied to the apical part of the insert (on Caco-2 cells) at a final concentration of 100 µg/ml. The positive control consisted of a co-culture treated with *E. coli* LPS, while the negative control group was a co-culture without any treatment. Additional controls included an insert with Caco-2 cells without PBMC, either untreated or treated with *E. coli* LPS, and untreated PBMC without the insert. After 4 days of exposure, the media were collected for further analyses, the Caco-2 cells were washed with ice-cold PBS and prepared for western blot, PBMC were collected and prepared for flow cytometry.

PBMC were treated with LPS at a final concentration of 1 µg/ml. Positive control was treated with *E. coli* LPS, and negative control was untreated. After 4 days of exposure, the medium was collected for further analyses, the cells were collected and prepared for flow cytometry.

Lactate dehydrogenase assay

80 µl of the sample was mixed with the reaction mixture in a ratio of 1:1 and incubated RT. The absorbance was measured at 492 nm using a SPECTRA Sunrise microplate reader (Tecan, Mannedorf, Switzerland). Untreated cells lysed by supplier-provided lysis buffer were used as a positive control.

Flow cytometry

The medium from PBMC was collected and centrifuged to capture all floating cells. PBMC in wells were incubated with 500 µl of 10 mM EDTA/PBS (10 min, 37 °C). Detached cells were moved to a flow cytometry tube. Another 500 µl of 10 mM EDTA/PBS was added to the wells, and any remaining cells were scraped with a cell scraper and moved to the tube with the rest of the respective sample. Cells were washed with PBS and blocked with 150 µl of PBS with 10% human serum (15 min, 4 °C). Human serum was a heat-inactivated pool of sera from 4 healthy donors prepared in our lab. Blocked cells were stained with antibodies against CD14 (APC anti-human CD14, 2109040) and CD16 (FITC anti-human CD16, 2110030, both SONY Biotechnology), 3 µl of each per sample (20 min, 4 °C). Stained cells were washed with cold PBS, the cells were re-suspended in 150 µl of cold PBS and kept on ice until measured on a flow cytometer (BD FACSVerse, BD Biosciences, USA). Immediately before measurement, 2 µl of propidium iodide (1 mg/ml) was added to the samples as a viability marker.

ELISA

Kits from Invitrogen and RaD Systems were used for the analyses. Invitrogen: Human CCL2 Uncoated ELISA Kit, Human IL-6 Uncoated ELISA Kit, Human IL-8 Uncoated ELISA Kit, Human IL-10 Uncoated ELISA Kit, Human TNFα Uncoated ELISA Kit; RaD Systems: Human CCL20 DuoSet ELISA. The absorbance was measured using a SPECTRA Sunrise microplate reader.

Western blot

The proteins were separated by SDS polyacrylamide gel electrophoresis using 7.5% gel (ZO-1), 10% gel (filaggrin, involucrin), 12.5% gel (claudin 4, occludin) or 15% gel (claudin 2). Primary antibodies against human ZO-1 (1:1000; 33-9100, Thermo Fisher Scientific), occludin (1:1000, sc-133256), claudin 2 (1:1000, sc-293233), claudin 4 (1:1000, sc-376643), filaggrin (1:500, sc-66192) and involucrin (1:200, sc-53361, all Santa Cruz Biotechnology, USA) were used. The total level of vinculin (1:1000, #13901), GAPDH (1:1000, #2118S, both Cell signalling technology), or β-actin (1:5000, sc-47778, Santa Cruz Biotechnology) was detected as a loading control. The secondary antibodies conjugated with HRP were diluted 1:2000, anti-mouse IgG (#7076) or anti-rabbit IgG (#7074, both Cell signalling technology), respectively. The immunoreactive bands were detected by an ECL detection reagent kit (Pierce, USA) using Amersham Imager 680 (GE HealthCare, USA). Relative protein levels were quantified by ImageJ software (Schneider et al., 2012, https://doi.org/10.1038/nmeth.2089), and the level of the protein of interest was normalized to the respective loading control.
